# *Roseiterribacter gracilis* gen. nov., sp. nov., a novel filterable alphaproteobacterium isolated from soil using a gel-filled microwell array device

**DOI:** 10.1371/journal.pone.0304366

**Published:** 2024-06-10

**Authors:** Ryosuke Nakai, Hiroyuki Kusada, Fumihiro Sassa, Ayaka Makino, Susumu Morigasaki, Hisayoshi Hayashi, Naoki Takaya, Hideyuki Tamaki

**Affiliations:** 1 Bioproduction Research Institute, National Institute of Advanced Industrial Science and Technology (AIST), Sapporo, Hokkaido, Japan; 2 Bioproduction Research Institute, National Institute of Advanced Industrial Science and Technology (AIST), Tsukuba, Ibaraki, Japan; 3 Department of Electronics, Graduate School of Information Science and Electrical Engineering, Kyushu University, Fukuoka, Japan; 4 Institute of Life and Environmental Sciences, University of Tsukuba, Tsukuba, Ibaraki, Japan; 5 Microbiology Research Center for Sustainability, University of Tsukuba, Tsukuba, Ibaraki, Japan; Institute of Biological Sciences, University of the Philippines Los Baños, PHILIPPINES

## Abstract

Our previous studies indicate the abundant and diverse presence of yet-to-be-cultured microorganisms in the micropore-filtered fractions of various environmental samples. Here, we isolated a novel bacterium (designated as strain TMPK1^T^) from a 0.45-μm-filtered soil suspension by using a gel-filled microwell array device comprising 900 microwells and characterized its phylogenetic and physiological features. This strain showed low 16S rRNA gene sequence identities (<91%) and low average nucleotide identity values (<70%) to the closest validly described species, and belonged to a novel-family-level lineage within the order *Rhodospirillales* of *Alphaproteobacteria*. Strain TMPK1^T^ exhibited small cell sizes (0.08–0.23 μm^3^) and had a high cyclopropane fatty acid content (>13%), and these characteristics were differentiated from other *Rhodospirillales* bacteria. A comprehensive habitability search using amplicon datasets suggested that TMPK1^T^ and its close relatives are mainly distributed in soil and plant-associated environments. Based on these results, we propose that strain TMPK1^T^ represents a novel genus and species named *Roseiterribacter gracilis* gen. nov., sp. nov. (JCM 34627^T^ = KCTC 82790^T^). We also propose *Roseiterribacteraceae* fam. nov. to accommodate the genus *Roseiterribacter*.

## Introduction

Soil microorganisms play a crucial role in the cycling of carbon and other nutrients and the regulation of plant growth [1, 2]. Recent extensive studies utilizing high-throughput sequencing technology have expanded our knowledge of soil microbial diversity; however, the vast majority of microorganisms, particularly archaea and bacteria, remain uncharacterized due to the difficulty of their isolation and cultivation [3, 4]. Therefore, the cultivation of uncultured microorganisms is essential to gain deeper insight into their ecological roles [5, 6]. Previous culture-independent analysis revealed that rare or poorly characterized bacterial taxa are present in the small cell-size fraction (<0.8 μm) of soil samples [7]. Additionally, we observed the presence of novel filterable and culturable microorganisms in the filtrates of soil suspensions filtered through micropore filters (0.22 μm or 0.45 μm filters), including the widely distributed *Terrihabitans* lineage [8–10]. However, our trial and other studies have also shown that culturable slender bacteria with small cell width can occasionally pass through filters (e.g., *Hylemonella* spp.) [8, 11, 12]. Such slender and fast-growing bacteria may preferentially form colonies on conventional agar plates and consequently impede the growth of slow growing and filterable bacteria. To overcome this challenge, fast-growing organisms considered as “contaminants” and small cell-sized bacteria must be cultivated in separate compartments (e.g., microwells).

The past decade has witnessed the development of novel cultivation methods that improve the culturability of difficult-to-culture microorganisms [13]. For instance, the isolation chip, widely-known as the “iChip,” with its array of multiple diffusion chambers, facilitates high-throughput *in situ* cultivation of novel soil microorganisms [14, 15]. This array further enabled us to inoculate one cell per chamber [16]. Recently, Sassa and colleagues developed a microwell array device that can separately culture microbial cells in multiple gel-filled microwells [17]. The surface of the microwells is covered with a water repellent, which prevents microbial cell migration in and out between the gel-filled wells. Unlike in the iChip-based diffusion system, microbial cells can be directly inoculated on the agar gel surface in the microwell array device, and the microcolonies grown therein can be picked and re-cultured. We have adopted this device for the cultivation of small soil microorganisms. During the proof-of-concept validation for the device, we successfully cultivated and isolated a novel 0.45-μm-filterable bacterium, designated as strain TMPK1^T^, from upland soil. In this study, we phylogenetically and physiologically characterize this strain, and based on the results we propose that strain TMPK1^T^ represents a novel genus and species named *Roseiterribacter gracilis* gen. nov., sp. nov. We also propose the novel taxon *Roseiterribacteraceae* fam. nov. to accommodate the genus *Roseiterribacter*.

## Materials and methods

### Preparation of the gel-filled microwell array device

A microwell array device composed of 900 microwells (device body 76 × 26 mm; well pitch, 1.0 mm; well size, 600 μm by 600 μm; well depth, 800 μm) was used for the cultivation of microbial cells. This array device was designed in accordance with a previous study [17]. All subsequent procedures described below were performed under ultraclean conditions (ISO class I) generated by Table KOACH with a flying debris prevention plate (KOKEN, Tokyo). Potential contamination with airborne microorganisms was monitored using a handheld particle counter (Model 3888; KANOMAX, Osaka, Japan). The device was coated with Fluorosurf (Fluoro Tech, Aichi, Japan) using sterile gauze to make the surface hydrophobic and dry, which can prevent cross-contamination of isolated microbial cells via the convexities between the miniature chambers. After coating, the device was placed in UltraPure DNase/RNase-free distilled water (Thermo Fisher Scientific, Waltham, MA) in a sterile glass Petri dish and then degassed using a vacuum desiccator (RVD-250; AS ONE Corporation, Osaka, Japan) and vacuum pump (DAP-6D; ULVAC, Kanagawa, Japan). After overnight immersion at room temperature (approximately 23°C), the device was washed by immersion in 70% ethanol and then placed in autoclaved 1/100-strength tryptic soy agar medium [18] (pH 6.0; hereafter denoted as 1/100 TSA) containing 0.17 g Bacto tryptone (Difco; Becton, Dickinson and Company, Franklin Lakes, NJ), 0.03 g Bacto soytone (Difco), 0.025 g glucose, 0.05 g NaCl and 0.025 g K_2_HPO_4_, and 8 g agar per 1 L of distilled water, in a glass Petri dish. The nutritionally diluted medium was used to isolate oligotrophic microorganisms in the environment and to suppress the overgrowth of fast-growing microorganisms against slow-growing ones. Prior to solidification of the agar gel, the Petri dish was degassed, the device was removed from the medium, the medium was allowed to solidify, and the excess gel was scraped off using an autoclaved polyimide film (AS ONE Corporation).

### Inoculation, cultivation, and isolation of filterable bacteria

A 150 g of soil sample was collected on December 25, 2019 in a sterilized sample bag from upland soils in plots fertilized with three macronutrients (nitrogen, phosphate, and potassium) at the Tsukuba-Plant Innovation Research Center (T-PIRC), University of Tsukuba, Ibaraki, Japan. The plot has a crop rotation of buckwheat, wheat, rye, groundnut, potato, and sweet potato [19], and rye was sprouting at the time of soil sampling. The sample was stored at 15°C until subsequent experiments. A 3 g aliquot of soil was suspended in 27 ml UltraPure DNase/RNase-free distilled water and then shaken at 150 rpm for 30 minutes. The resulting suspension was filtered through a sterile syringe filter with a 0.45-μm pore size (Millex-HV syringe filter unit; Merck Millipore, Burlington, MA). The filtered suspension (50 μl) was spread onto a gel-filled microwell array device prepared using a sterile cell scraper (Sumitomo Bakelite, Tokyo). Following the removal of excess suspension using sterile gauze (Yamato, Osaka, Japan), the device lid was attached to the microwells, and the four corners of the device body were sealed with Kapton tape. After incubation for 6 days at 25°C, the presence or absence of the microcolony formation on each microwell was observed with a stereoscopic microscope (Stemi 305; Carl Zeiss AG, Oberkochen, Germany) equipped with a color camera (Axiocam 208 color; Zeiss). The microcolonies were picked with a sterile 10 μL barrier pipette tip (Thermo Fisher Scientific) and then transferred onto a conventional 1.5% agar plate containing the same ingredients as those used to fill the device (i.e., 1/100 TSA). Single-colony picking was repeated thrice to ensure colony purification. For comparison, a conventional agar medium with the same ingredients (1/100 TSA) was prepared and used to screen for filterable bacteria. Screening with conventional, undiluted standard TSA (Difco) and R2A (Nihon Pharmaceutical, Tokyo) media was also performed.

### Phylogenetic and phylogenomic analysis

The genomic DNA of the purified isolates was extracted using a NucleoSpin Microbial DNA Kit (TaKaRa Bio, Shiga, Japan) following the manufacturer’s protocol. PCR amplification of the 16S rRNA genes was performed with the universal primers 10F (5′-AGAGTTTGATCMTGGCTCAG-3′) and 1492R (5′-TACGGYTACCTTGTTACGACTT-3′), *Ex Taq* polymerase, and accompanying reagents (TaKaRa Bio). PCR products were sequenced using an Applied Biosystems BigDye Terminator v3.1 Cycle Sequencing Kit (Thermo Fisher Scientific) with the sequencing primer 907R (5′-CCGYCAATTCMTTTRAGTTT-3′) and an Applied Biosystems 3500xL Genetic Analyzer (Thermo Fisher Scientific). The obtained partial sequences were compared with known sequences in the EzBioCloud database (https://www.ezbiocloud.net/) using a BLAST search and pairwise sequence alignment [20–22]. The novel candidate strain TMPK1^T^ was selected and used for subsequent analyses. The full-length 16S rRNA gene sequence (1482 bp) of TMPK1^T^ was retrieved from a genome sequence obtained in a previous study (DDBJ/ENA/GenBank accession nos. BOPV01000001.1 to BOPV01000003.1) [23]. It should be noted that the near full-length 16S rRNA gene sequence of the strain was also obtained by Sanger sequencing with sequencing primers 10F, 787F (5′-ATTAGATACCCNGGTAG-3′), 907R, 909F (5′-ACTYAAAKGAATTGRCGGGG-3′), and 1492R and confirmed to be a complete match to that recovered from the genome. To identify the closest type strains, uncultivated clones/phylotypes, and invalidly described strains, the sequence was searched against the NCBI nt/nr database using BLAST [20]. The sequence of strain TMPK1^T^ was aligned with related sequences using ClustalW [24]. Based on the alignment of the 16S rRNA gene sequences, a phylogenetic tree was constructed using the neighbor-joining method [25] with MEGA7 [26]. The evolutionary distances of the tree were computed using the Jukes-Cantor model [27] with the bootstrap test (1000 replicates) [28]. Bootstrap values (100 replicates) were also computed using maximum-likelihood method. For further genome-wide phylogenetic analysis, this study followed the taxonomy of the genome taxonomy database (GTDB; https://gtdb.ecogenomic.org/) [29, 30], and a phylogenomic placement was examined. A phylogenomic tree of the class *Alphaproteobacteria* was generated using phyloT v2 (https://phylot.biobyte.de/) with GTDB release 207 (R207), in which the TMPK1^T^ genome was incorporated. The GTDB taxonomy was inferred using FastTree [31] based on 120 single copy marker proteins [30]; GTDB R207 was found to contain 317,542 genomes organized into 65,703 species clusters (see the following website for details on the R207 statistics: https://gtdb.ecogenomic.org/stats/r207). The tree obtained was visualized using iTOL v6 [32].

### Morphological, physiological, and biochemical characterization

After isolation by a gel-filled array device, strain TMPK1^T^ was routinely cultivated in R2A agar (Nihon Pharmaceutical) at 25°C under light (light condition: 16h/day photoperiod at approximately 10,000 lux provided by a BR-53FP incubator equipped with an LC-LED-450W; TAITEC, Saitama, Japan). Unless otherwise stated, cells cultured under these conditions were used in subsequent experiments. R2A medium was used because the growth of this strain was higher in R2A under light than in the 1/100 TSA used for initial isolation. Cell morphology was examined under a microscope (Olympus BX-50F4; Olympus Optical, Tokyo). Cell volume was calculated according to a previous report [33]. Cell motility was determined by using an Axio Observer 7 inverted microscope (Carl Zeiss, Oberkochen, Germany). To further observe the presence or absence of flagella or flagellum-like structures, cells were observed by a scanning electron microscope (SEM). Briefly, fixed cells were dehydrated in a series of ethanol solutions (50%, 70%, 90% and 100%), substituted with tert-butyl alcohol, vacuum freeze-dried, coated with a thin layer (30 nm) of osmium by an NL-OPC80A osmium plasma coater (Nippon Laser & Electronics Lab., Nagoya, Japan), and finally, observed under SEM (JSM-7500F; JEOL Ltd., Tokyo) at an acceleration voltage of 3.0 kV. Spore formation was examined by microscopic observation of cells at different incubation times and after heat treatment at 80°C for 10 minutes. Gram staining was performed using a Favor G Nissui kit (Nissui Pharmaceutical). Growth was assessed by R2A agar plate count at a range of temperatures (10, 15, 20, 25, 30, 37, 40, and 45°C). Growth at various pH levels (pH 5.0, 5.5, 6.0, 6.5, 7.0, 7.5, 8.0, and 8.5) and salt concentrations (0, 0.2, 0.4, 0.6, 0.8, 1, 2, 3, 4, and 5% [w/v] NaCl) was also tested with R2A agar. Anaerobic growth was examined using a chamber with an Anaero Pack system (Mitsubishi Gas Chemical, Tokyo). Catalase and oxidase activities were tested as described in a previous study [34]. Physiological and biochemical characteristics and enzyme activities were evaluated with API 20 NE and API ZYM test strips (bioMérieux, Marcy-l’Étoile, France). Lipase activity was determined by precipitation around the colonies grown on R2A agar supplemented with 1% Tween 20, Tween 40, Tween 60, or Tween 80.

### Chemotaxonomic and pigment analyses

Chemotaxonomic analysis was performed as previously reported [10]. Briefly, the cellular fatty acids of strain TMPK1^T^ were determined using a Sherlock Microbial Identification system version 6.0 (Microbial ID; MIDI Inc., Newark, DE) and the TSBA library database (TSBA6 6.20). The major quinones were analyzed using an ultra-performance liquid chromatography (UPLC) system (Acquity UPLC H-Class system; Waters, Milford, MA) equipped with a photodiode array detector (UPPDA-E; Waters), an Eclipse plus C18 column (2.1 × 150 mm; pore size, 1.8 μm; Agilent Technologies, Santa Clara, CA), and MassLynx V4.2 software (Waters). Polar lipids were characterized using two-dimensional HPTLC silica gel 60 (Merck Millipore). The genomic G+C content was calculated from the genome sequence of strain TMPK1^T^ (accession nos. BOPV01000001.1 to BOPV01000003.1). Pigments were extracted with acetone:methanol:50 mM Tris-HCl (pH 7.0) (7:2:1, v/v), and their absorption spectra were recorded with an Acquity UPLC H-Class system (Waters), MassLynx V4.2 software (Waters), and an Acquity BEH C18 column (2.1 × 150 mm; pore size, 1.7 μm; Waters).

### Tests for plant growth-promoting properties

To investigate the potential symbiotic effects of strain TMPK1^T^ on plants, five properties typically found in terrestrial plant growth-promoting bacteria (PGPB) were tested: indole-3-acetic acid (IAA) production, siderophore production, phosphate solubilization, phosphate release potential, and nitrogen fixation potential. These assays were performed using cells grown in R2A, as reported previously [35]. Briefly, IAA production was tested by Salkowski’s method, with the supplemental addition of l-tryptophan (as a precursor of IAA); the IAA standard was prepared as 20, 10, 5, and 1 μg mL^−1^; cells grown in R2A liquid without light were used to determine pink coloration. Siderophore production was tested using chrome azurol S (CAS) agar medium [36]. Three types of CAS agar were prepared: CAS, CAS cut in half and replaced with R2A agar, and CAS overlaid with R2A agar. Phosphate solubilization was evaluated using Pikovskaya medium (called PVK), National Botanical Research Institute’s phosphate growth medium (NBRIP), and National Botanical Research Institute’s phosphate growth medium devoid of yeast extract (NBRIY) agar media [37]. The potential for phosphorus release and nitrogen fixation was examined with the presence or absence of genes encoding key enzymes (i.e., nitrogenase) in the annotated TMPK1^T^ genome according to a previous study [38]. The presence of genes involved in IAA production, phosphate solubilization, and siderophore production was also examined following the previous study [38].

### Potential habitability analysis

The potential habitability of strain TMPK1^T^ and its close relatives was investigated using the integrated microbial next generation sequencing (IMNGS), a database search against integrated 16S rRNA gene amplicon datasets [39]. Using the 16S rRNA gene sequence of strain TMPK1^T^ as a query sequence, IMNGS was performed with sequence similarity thresholds of 97% and 99%. Habitat preference was evaluated using ProkAtlas, which contains >360,000 16S rRNA gene sequences labeled by one environmental category [40]. Similar to IMNGS, the TMPK1^T^ 16S rRNA gene sequence was used as a query and searched at a 97% similarity threshold.

## Results and discussion

### Isolation and cultivation of a novel filterable bacterium

**Fig 1:**
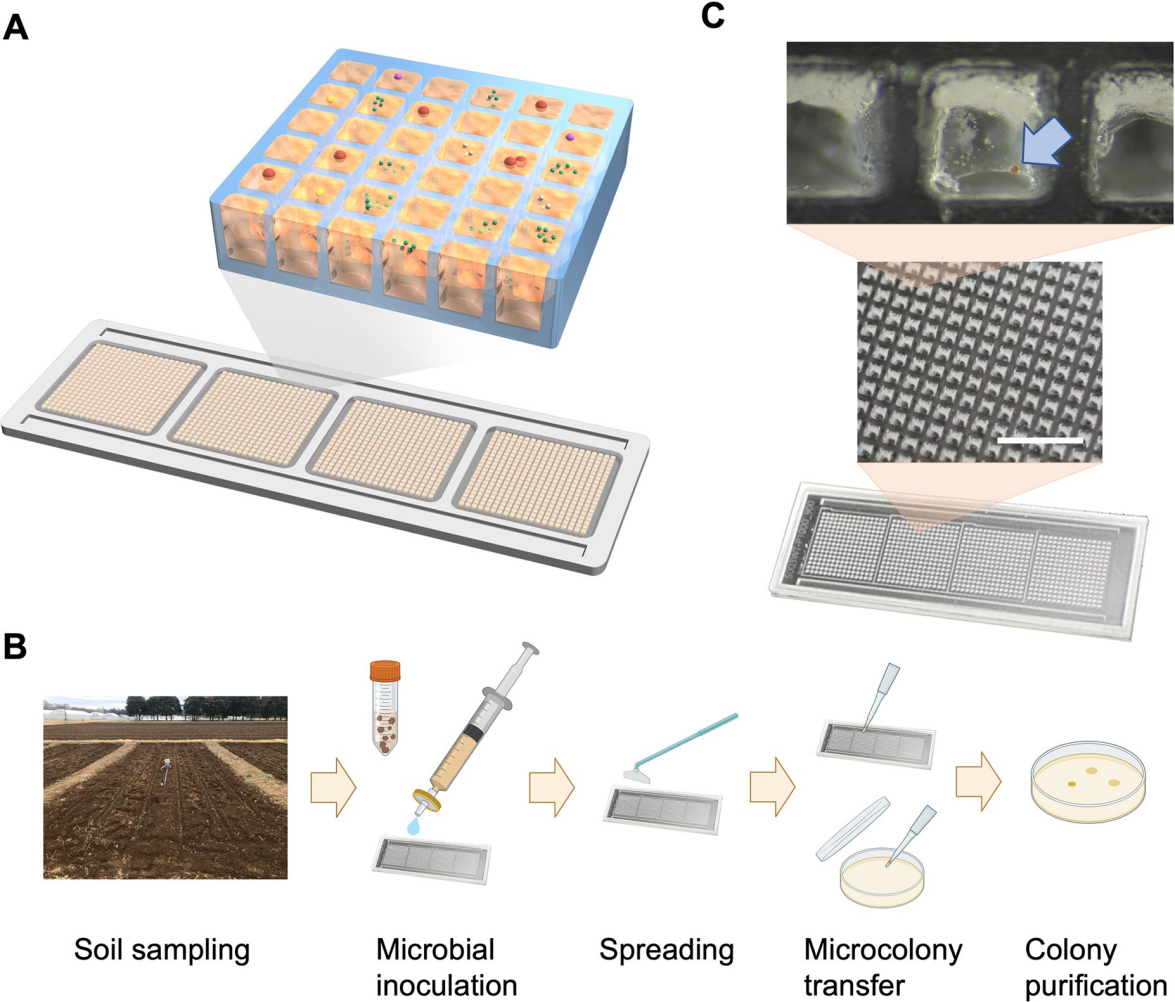
Cultivation of microorganisms using a gel-filled microwell array device. (**A**) Schematic representation of the cultivation of microbial cells on microwells. (**B**) Image of upland soil in the study area, and illustration of the inoculation, cultivation, and isolation procedures of filterable soil microorganisms in the microwell array device; the schematic illustration was created with BioRender (https://biorender.com/). (**C**) The microwell array device (device body 76 × 26 mm; 900 microwells; well pitch, 1.0 mm; well size, 600 μm by 600 μm; well depth, 800 μm) (*bottom*) and close-up images of its microwells; scale bar, 5 mm (*middle* and *upper*); microcolonies formed on the gel in the microwell after 6 days of cultivation at 25°C (*light blue arrow*).

Using a gel-filled microwell array device, strain TMPK1^T^ was isolated from a 0.45-μm-filtered suspension of upland soil (**Fig 1A–1C**). The array device used in this study comprised 900 microwells, each filled with 1/100 TSA medium. Filterable microorganisms persisting in the suspension formed more than 100 microcolonies on the medium after 6 days at 25°C (**Fig 1C**). At the time of observation, >95% of the agar gels in the microwells showed neither distinct evaporation nor associated gel deformation. Ten microcolonies were picked randomly from the gel-filled microwells, and then transferred and purified by single colony picking onto conventional agar plates containing the same ingredients (i.e., 1/100 TSA).

Of the 10 isolates purified from 10 microwells, 9 isolates showed high similarity of the partial 16S rRNA gene sequence (99–100%) to the known type strains belonging to *Bradyrhizobium* and *Polaromonas*, while one isolate, designated TMPK1^T^, had a lower identity (<91%) and was then selected for further characterization (see details below); strain TMPK1^T^ formed only a small number of colonies on two conventional agar plates—1/100 TSA and TSA media—but showed good growth on the R2A medium (**S1 Fig in S1 Appendix**). R2A medium was used for subsequent characterization.

In comparative experiments with the conventional agar plates (1/100 TSA, TSA, and R2A media) without the array device, the tens of isolates were closely related to the known genera *Bradyrhizobium*, *Caulobacter*, *Mesorhizobium*, and *Polaromonas*. No isolates related to strain TMPK1^T^ were isolated by the standard agar plate culture method. Thus, the gel-filled microwell device used in this study was at least successful in isolating strain TMPK1^T^. Although the practicality of this method needs to be further evaluated, an approach using multiple chambers may be suitable for culturing certain novel bacteria, including micropore-filterable bacteria, through separation from other fast-growing bacteria.

### Phylogenetic affiliation of strain TMPK1^T^ and its close relatives

Based on the 16S rRNA gene sequence comparisons, strain TMPK1^T^ was affiliated with the class *Alphaproteobacteria* of the phylum *Pseudomonadota* (renamed from *Proteobacteria* [41]) but showed low sequence identities (<91%) with closely related type strains, such as *Skermanella pratensis* W17^T^ (identity 90.69%; BLAST total score of 12297) [42] and *Haematospirillum jordaniae* H5569^T^ (90.87%; 10258) [43], within the family *Rhodospirillaceae* of the order *Rhodospirillales*. The genome sequence of strain TMPK1^T^ (DDBJ/ENA/GenBank accession no. BOPV01000001.1–BOPV01000003.1 [23]) also showed low average nucleotide identity (ANI) values (<70%) compared with those of the type strains. The 16S rRNA gene-based phylogenetic tree suggested that strain TMPK1^T^ is a representative of a novel family along with one non-validly described bacterium, SC-11 (accession no. LC602157) (**S2 Fig in S1 Appendix** and **S1 Table in S2 Appendix**). The SC-11 bacterium is described in the DDBJ/ENA/GenBank database as being isolated using an electrode system that likely differs from the conventional plating method; however, the details are unpublished.

**Fig 2:**
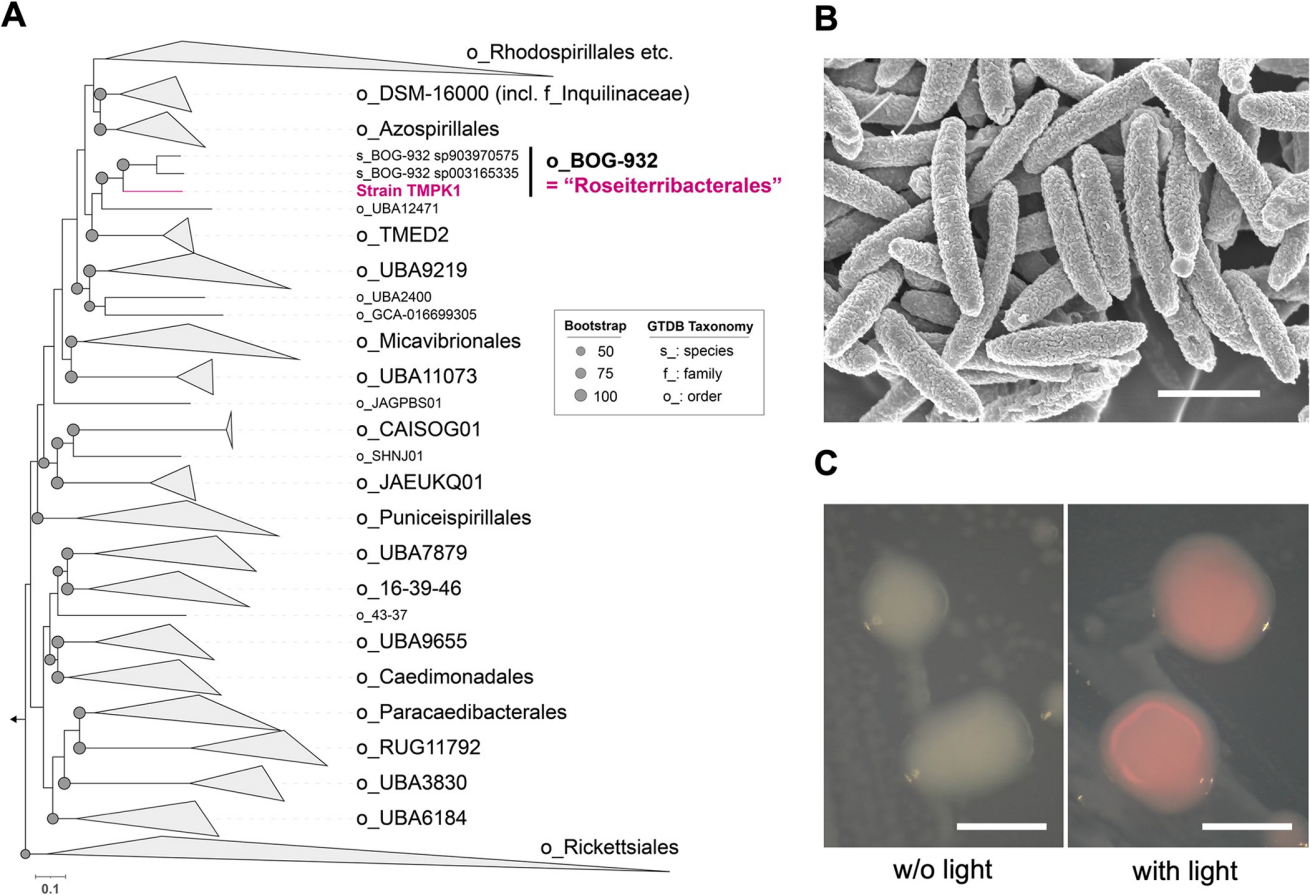
Genome-based phylogeny, cell morphology, and colonies of strain TMPK1^T^. (**A**) Phylogenomic placement of strain TMPK1^T^ and members of the order *Rhodospirillales* and other orders based on GTDB release 207. (**B**) Scanning electron micrograph of cells of strain TMPK1^T^; scale bar, 1 μm. (**C**) Color of colonies of strain TMPK1^T^; scale bar, 0.5 mm.

We examined a phylogenomic placement based on concatenated taxonomic marker gene sequences following the GTDB-based taxonomy (**Fig 2A**) [29]. This tree indicated that strain TMPK1^T^ was the first cultivated representative of the BOG-932 lineage, which is an order-level lineage, together with two metagenome-assembled genomes (MAGs) in the GTDB. These two MAGs of uncultivated bacteria were recovered from the permafrost active layer soil (GCA_003165335.1 [44]) and alpine bog (GCA_903970575.1), respectively; note that the order level is named after the MAG identifier (bog_932) of the former permafrost soil. The GTDB-based tree also showed that the BOG-932 lineage, including TMPK1^T^, is adjacent to “Azospirillales”, which corresponds to *Rhodospirillales* in the NCBI taxonomy. “Azospirillales” includes members of the genus *Azospirillum*, well-studied PGPB [45, 46]. Discrepancies between 16S rRNA gene-based and phylogenomic trees have been found in the analysis of other alphaproteobacterial members, mainly due to insufficient taxon sampling and under- or overestimated branch support [47]. At the time of writing, the number of MAGs closely related to strain TMPK1^T^ is very limited in the GTDB. Collectively, these results suggested that a novel family should at least be created for strain TMPK1^T^.

### Morphological, physiological, and biochemical characteristics of strain TMPK1^T^

Cells of strain TMPK1^T^ were rods, with a size of 0.3–0.4 × 1.3–2.0 μm and a volume of 0.08–0.23 μm^3^ (**Fig 2B**). These smaller cells were as small as typical ultramicrobial cells, which are defined by a cell volume of less than 0.1 μm^3^ [12]. Colonies of TMPK1^T^ grown on R2A agar were circular, white, convex, and <1 mm in diameter after 72 h incubation at 25°C. The colonies changed from white to pink in the presence of light (**Fig 2C**), and there was no remarkable difference in their growth with or without light. We further confirmed that the absorption peaks of pigment of the cells cultured under the light condition were approximately 500 nm (**S3 Fig in S1 Appendix**), indicating the presence of carotenoids [48]. This finding was consistent with that of our previous investigation to detect the carotenoid biosynthesis genes (e.g., the phytoene desaturase gene) in the TMPK1^T^ genome [23]. Other *Rhodospirillales* bacteria have also been reported to contain carotenoid pigments [49], but to our knowledge, pigment production induced by light is rare among *Rhodospirillales* members.

Strain TMPK1^T^ was Gram-negative, catalase-negative, and oxidase-positive. Motility was observed by microscopy. Some cells with flagellum-like structures, possibly related to motility, were also observed using scanning electron microscopy (**S4 Fig in S1 Appendix**). This strain did not grow anaerobically. Growth was observed at 15°C–37°C, but not at 10°C or 40°C, and at pH 5.5–8.0. No or weak growth was observed in the presence of >0.6% NaCl. All API 20NE tests were negative. In the API ZYM tests, alkaline phosphatase, esterase (C4), esterase lipase (C8), leucine arylamidase, valine arylamidase, trypsin, acid phosphatase, and naphthol-AS-BI-phosphohydrolase were positive, whereas all others were negative. This strain hydrolyzed Tween 40 by lipase production (**S5 Fig in S1 Appendix**). Details on the enzymatic activities of API ZYM are included in the species description.

### Chemotaxonomic characteristics of strain TMPK1^T^

The major cellular fatty acids (>3% of the total cellular fatty acids) of strain TMPK1^T^ were summed feature 8 containing C_18 : 1_ω7*c* and/or C_18 : 1_ω6*c* (49.5%), C_19 : 0_ cyclo ω8*c* (13.4%), summed feature 2 containing C_16 : 1_*ω*7c and/or C_16 : 1_*ω*6c (9.72%), C_16:0_ 3-OH (8.2%), summed feature 3 containing C_16:1_*ω*7*c* and/or C_16:1_*ω*6*c* (6.1%), and C_16:0_ (4.8%); other minor fatty acids were C_18:1_ 2-OH (2.6%) and C_19:0_ 10-methyl (2.0%). Cyclopropane fatty acids, such as C_19 : 0_ cyclo, in the cell membrane are known to play a key role in tolerance to environmental stresses, such as high temperature, low pH, and low aeration [50, 51]. The physiological impact of cyclopropane fatty acids on the TMPK1^T^ cell membrane remains unclear, but as described below, a high content of this fatty acid is rare among related type strains and may be a potential marker to discriminate this lineage from other genera or families. The quinone in the strain was ubiquinone-10 (Q-10). The dominant polar lipids detected were phosphatidylglycerol and unidentified amino lipids, whereas the other minor lipids were diphosphatidylglycerol, phosphatidylcholine, three unidentified polar lipids, unidentified amino lipids, and unidentified amino phospholipids (**S6 Fig in S1 Appendix**). The genomic G + C content was 63.7% as calculated from the TMPK1^T^ genome sequence. Slightly-high to high G+C contents (i.e., 60%–70%) are also observed in other *Rhodospirillales* members (from the data in Bac*Dive* [52]).

### Plant growth-promoting potential of strain TMPK1^T^

Strain TMPK1^T^ was obtained from upland soil and placed relatively close to “Azospirillales,” which includes well-known PGPB species, in a GTDB-based tree. Therefore, we examined five plant growth-promoting traits of PGPB in this strain: indole-acetic acid (IAA) production, siderophore production, phosphate solubilization, phosphate release potential, and nitrogen fixation potential. The IAA production of 18.9 μg mL^−1^ was experimentally confirmed under the condition using tryptophan as a precursor for IAA biosynthesis. The TMPK1^T^ genome possessed some of the genes encoding enzymes, including monoamine oxidase (locus tag TMPK1_10690 in the NCBI database), which may be involved in IAA production. Siderophore production and phosphorus solubilization activities were negative under the conditions tested. Consistent with these findings, the *ent* and *pvd* genes, which are involved in the biosynthesis of typical siderophores, such as enterobactin and pyoverdine, were not found in the TMPK1^T^ genome. Key genes involved in phosphorus solubilization, particularly those involved in gluconic acid and alpha-ketogluconic acid metabolism, were also not found in the genome. No genes involved in nitrogen fixation were identified (e.g., *nifH*, *nifD*, and *nifK*). In contrast, the results of gene annotations revealed the presence of a gene cluster related to phosphate release via phosphonate degradation (e.g., *phnM*, *phnL*, *phnK*, *phnF*, *phnE*, *phnD*, and *phnC*; locus tags in the NCBI, from TMPK1_02150 to TMPK1_02260). Note that such a gene cluster was not found in the two related MAGs mentioned above (at least based on the annotation of genes in the metagenome-derived fragmented MAGs). Together, these results indicate that strain TMPK1^T^ possessed IAA production capacity and phosphate release potential as common traits with well-known PGPBs [53, 54]. Given that this strain is phylogenetically novel at the family level, further studies might lead to the elucidation of unforeseen mechanisms mediating plant–microbe interactions.

The soil plot from which strain TMPK1^T^ was isolated has a crop rotation of buckwheat, wheat, rye, groundnut, potato, and sweet potato [19], and rye was cultivated at the time of soil collection in this study. Although the contribution of the strain to the growth of rye and other crops was not investigated in this study, enhancement of germination capacity and seedling growth by *in vitro* treatment of rye seeds with IAA-producing bacteria has been reported [55]. Future studies to examine the growth effect under *in vitro* or pot culture conditions as well as the changes in the abundance and gene expression (e.g., growth promotion-related genes) of TMPK1^T^ during crop rotation will provide better answers regarding the importance of this strain in the rhizosphere.

### Potential distribution and habitat of strain TMPK1^T^ and its relatives

Based on the IMNGS search, which is a database search against integrated 16S rRNA gene amplicon datasets [39], we found that TMPK1^T^ matched 2698 datasets at a >97% similarity threshold, with its source environments being mainly soil (1841 datasets), plants (257), rhizosphere (169), the subalpine forb *Boechera stricta* (75), and freshwater (68); at a higher threshold of >99%, it matched 824 datasets derived from soil (623), plants (68), rhizosphere (30), and freshwater (24). Through further database searches using ProkAtlas containing >360,000 16S rRNA gene sequences labeled by one environmental category [40], the potential habitat preferences of TMPK1^T^ and its relatives were found to be soil (score, 25.9%), rhizosphere (13.2%), biofilm (6.0%), freshwater (5.4%), ground water (4.6%), and rock pore water (3.5%). These results suggest that the novel lineage containing TMPK1^T^ is mainly distributed in soil and plant-associated environments. Mining the micropore-filtered fractions of environmental samples would likely capture the presence of close relatives.

### Proposal of a novel family, genus, and species for strain TMPK1^T^

The novel filterable bacterium strain TMPK1^T^ showed low 16S rRNA gene sequence identities (<91%) and ANI values (<70%) to the closely related type strains belonging to the order *Rhodospirillales*. Phenotypic differences were also observed in the closest strain, *S*. *pratensis* W17^T^, isolated from soil (**Table 1**; [42]). Moreover, strain TMPK1^T^ possesses features, including small cell size and high cyclopropane fatty acid (C_19 : 0_ cyclo ω8*c*) content (>13%), although the strain and its related *Rhodospirillales* members share several common traits: major fatty acid (i.e., C_18 : 1_*ω*7*c* or C_18 : 1_*ω*6*c*), ubiquinone-10, and relatively high GC content (**Table 2**; [42, 56–68]). The PGP traits and habitat preferences suggested that this strain is associated with plants and their growth, although further studies will be needed to confirm this. Based on these distinctive phenotypic features and the phylogenetic/phylogenomic placement, we propose the name *Roseiterribacter gracilis* gen. nov. and sp. nov. for strain TMPK1^T^. We further propose that the novel taxon *Roseiterribacteraceae* fam. nov., to accommodate the genus *Roseiterribacter*.

**Table 1:** Phenotypic characteristics that differentiate strain TMPK1^T^ from the closest type strain *Skermanella pratensis* W17^T^.

Characteristic	1	2
Isolation source	0.45-μm-filtered suspension of upland soil	meadow soil
Cell shape	small rods or rods	small rods
Cell size (μm)	0.3–0.4 × 1.3–2.0	0.7–1.2 × 1.1–1.5
Growth at/in presence of:		
10°C	−	+
pH 6	+	−
1.0% (w/v) NaCl	−	+
Enzymatic activities:		
Catalase	−	+
α-glucosidase	−	+
Trypsin	+	−
naphthol-AS–BI-phosphohydrolase	+	−

Strains: 1, strain TMPK1^T^ (this study); 2, *Skermanella pratensis* W17^T^ (Guo *et al*., 2020); +, positive; −, negative; Both strains are Gram-stain-negative, aerobic, and positive for oxidase; Both strains are positive for alkaline phosphatase, esterase (C4), esterase lipase (C8), leucine arylamidase, valine arylamidase, and acid phosphatase.

**Table 2:** Differential characteristics of strain TMPK1^T^ and representative genera and species in the families of the order *Rhodospirillales*.

Characteristic	1	2	3	4	5	6	7	8	9	10	11	12	13	14
Isolation source	0.45-μm-filtered suspension of upland soil	meadow soil	flowers, fruits, palm wine, vinegar, kefir, and fermented foods	soil, roots, stems, leaves, and seeds	biofilter of a marine aquaculture system	marine macroalga	freshwater river	freshwater	saltern	soil	seawater	coastal seawater	seawater	river water, river mud
Cell shape	small rods or rods	small rods	ellipsoidal to rods	plump, slightly-curved and straight rods	coccid	spiral (occasionally rod or filamentous)	rods	vibrioid to spiral	rod to spiral	six-pronged stars	s-shaped	slightly curved and straight rod	vibrioid to spiral	rod or straight to curved rod
Cell size (μm)	0.3–0.4 × 1.3–2.0	0.7–1.2 × 1.1–1.5	0.6–0.9 × 1.0–4.0	0.6–1.7 × 2.1–3.8	1.5–2.1	0.5–0.6 × 2.5–5.0	0.8 × 1.4–1.6	1.5–2.5 × 7–10	0.8–0.9 × 1.0–3.5	0.7–3.0	0.5–0.6 × 2.0–3.0	0.3–0.5 × 1.3–1.5	0.6 ×3–5	0.6–0.9 × 0.5–2.2
Motility	+	ND	+/−	+	−	+	−	+	+	−	+	+	+	+
Catalase	−	+	+*	+/−	+	+	−	ND	ND	+	−	+	+	+
Oxidase	+	+	−	+	ND	+	+	ND	ND	+	+	+	+	+
Temperature range for growth (°C) (optimum)	15–37	10–40	(30)	(33–41)	15–45	4–40	18–37	(30–35)	20–45	(28–30)	10–40	10–35	4–40	5–35
pH range (optimum)	5.5–8.0	7–8	(4.0–6.0)	(5.5–7.5)	5.5–11.0	3.5–9.5	ND	6.0–8.5	(7.5–8.0)	(neutral)	5.5–9.5	7–9	ND	6.0–8.0
NaCl range	0–0.4	0–2.0	ND	0–3.0	0.25–5.0^†^	0.3–10^†^	ND	ND	3–24	up to 1	1–6	1–10	up to 10	0–2.0
Major fatty acids (>10% of the total cellular fatty acids)	C_18 : 1_*ω*7*c* and/or C_18 : 1_*ω*6*c* (49.5), C_19 : 0_ cyclo *ω*8*c* (13.4)	C_18 : 1_*ω*7*c* and/or C_18 : 1_*ω*6*c*(48.5), C_16: 0_ (20.9), C_18: 0_ (14.9)	C_18 : 1_*ω*7	ND	ND	C_18 : 1_*ω*7*c* (48.6), C_16 : 1_*ω*7*c* (30.7)	ND	C_18:1_ (54.8), C_16:1_ (27.1), C_16:0_ (14.0)	C_18:1_ (35.2), C_18:0_ (23.0)	ND	C_18 : 1_*ω*7*c* (60.2), C_16:0 _ (13.4), C_16 : 1_*ω*7*c* and/or C_16 : 1_*ω*6*c* (11.1)	C_18 : 1_*ω*7*c* (48.5), C_16:0_ (14.8), C_17:0_ (12.2)	C_18 : 1_*ω*7*c* (45), C_16:0_ (16), C_16 : 1_*ω*7*c* (16)	C_18 : 1_*ω*7*c* and/or C_18 : 1_*ω*6*c* (34.6–44.0), C_18:1_ 2-OH (11.4–11.8), C_16:0 _ (9.6–13.3)
Quinone	Q-10	Q-10	Q-9	ND	ND	ND	ND	Q-10, RQ-10	Q-10, MK-10	ND	Q-10	Q-10	ND	Q-10
Major polar lipids	PG, UAL	DPG, PG, PE, PC	ND	ND	ND	ND	ND	ND	ND	ND	PE, PG, DPG, UAL, UPL, 4UL	ND	ND	DPG, PL, UAL, UGL, UL
G+C content (%)	63.7	67.3	50.5–60.3	64–71	60.3	51.1	ND	63.8–65.8	67.4	69.3–72.9	48.3	68.0	54.7	67.2–68.1

* Usually catalase positive except for *A*. *peroxydans* IFO13755^T^.

^†^ Data for artificial sea salts.

Taxa: 1, strain TMPK1^T^ (this study); 2, *Skermanella pratensis* W17^T^ (Guo *et al*., 2020); 3, *Acetobacter* (Sievers & Swings, 2015); 4, *Azospirillum* (Baldani *et al*., 2015); 5, *Geminicoccus roseus* D2-3^T^ (Foesel *et al*., 2007); 6, *Kiloniella laminariae* LD81^T^ (Wiese *et al*., 2009); 7, *Reyranella massiliensis* 521^T^ (Pagnier *et al*., 2011); 8, *Rhodospirillum rubrum* S1^T^ (Imhoff, 2015a); 9, *Rhodovibrio salinarum* DSM 9154^T^ (Imhoff, 2015b); 10, *Stella humosa* DSM 5900^T^ (Vasilyeva, 2015); 11. *Terasakiella salincola* KMU-80^T^ (Yoon & Kang 2018); 12, *Thalassobaculum litoreum* CL-GR58^T^ (Zhang *et al*., 2008); 13, *Thalassospira lucentensis* QMT2^T^ (López-López *et al*., 2002); 14, *Zavarzinia* (Meyer *et al*., 1993; Lee *et al*., 2019); +, positive, −, negative, +/−, positive or negative; ND, no data available; PG, phosphatidylglycerol; UAL, unidentified aminolipid; DPG, diphosphatidylglycerol; PE, phosphatidylethanolamine; PC, phosphatidylcholine; UPL, unidentified phospholipid; UL, unidentified lipid, UGL, unidentified glycolipid.

#### Description of *Roseiterribacter* gen. nov

*Roseiterribacter* (Ro.sei.ter.ri.bac’ter. L. masc. adj. *roseus*, rose/pink; L. fem. n. *terra*, earth; N.L. masc. n. *bacter*, a rod; N.L. masc. n. *Roseiterribacter*, pink-colored earth [soil] rod).

Cells are small, rod-shaped, and grow chemoorganotrophically and aerobically. Gram-negative, motile, non-spore-forming. Produces pink-pigmented colonies. The major cellular fatty acids are C_18 : 1_*ω*7*c* and/or C_18 : 1_*ω*6*c*, C_19 : 0_ cyclo *ω*8*c*, and C_16 : 1_*ω*7c and/or C_16 : 1_*ω*6c. The respiratory quinone was ubiquinone-10 (Q-10). Polar lipids include phosphatidylglycerol and unidentified amino-lipids.

This genus belongs to the family *Roseiterribacteraceae*. The type species is *Roseiterribacter gracilis*.

#### Description of *Roseiterribacter gracilis* sp. Nov

*Roseiterribacter gracilis* (gra’ci.lis. L. masc. adj. *gracilis*, slender, referring to slender cells of the type strain).

General descriptions of its morphological and chemotaxonomic features are provided in the description of the genus. Cells grown in R2A liquid medium are small and rod-shaped with a size of 0.3–0.4 × 1.3–2.0 μm and a volume of 0.08–0.23 μm^3^. Colonies on the R2A agar medium were circular, white, convex, and <1 mm in diameter. The colony color changed from white to pink under light conditions. The temperature range for growth on R2A is 15°C to 37°C. The pH range for growth is pH 5.5–8.0 (note that potential changes in pH during cultivation can affect the pH growth range data). The NaCl concentration for growth ranges from 0% to 0.4%. No or weak growth was observed in the presence of 0.6% NaCl. Catalase-negative and oxidase-positive. Enzymatic activities are positive for alkaline phosphatase, esterase (C4), esterase lipase (C8), leucine arylamidase, valine arylamidase, trypsin, acid phosphatase, and naphthol-AS-BI-phosphohydrolase and negative for lipase (C14), cystine arylamidase, chemotrypsin, α-galactosidase, β-galactosidase, β-Glucuronidase, α-glucosidase, β-glucosidase, *N*-acetyl-β-glucosaminidase, α-mannosidase, and α-fucosidase. Tween 40 hydrolysis activity is positive.

The type strain, TMPK1^T^ (= JCM 34627^T^ = KCTC 82790^T^), was isolated from the 0.45 μm-filtered filtrates of a suspension of upland soil from Tsukuba, Ibaraki, Japan. The DNA G+C content was approximately 64%. The genome is approximately 4.2 Mbp. The genome sequence was deposited in DDBJ/ENA/NCBI under accession nos. BOPV01000001.1 to BOPV01000003.1.

#### Description of *Roseiterribacteraceae* fam. Nov

*Roseiterribacteraceae* (Ro.se.i.ter.ri.bac.te.ra.ce’ae. N.L. neut. n. *Roseiterribacter* type genus of the family; suff. *-aceae*, ending to denote a family; N.L. fem. pl. n. *Roseiterribacteraceae*, the family of the genus *Roseiterribacter*).

The description is the same as for the genus *Roseiterribacter*.

This family belongs to the order *Rhodospirillales*. The type genus is *Roseiterribacter*.

## Supporting information

S1 Appendix (PDF)

S2 Appendix (XLSX)
