# Supplementary material for: Roseiterribacter gracilis gen. nov., sp. nov., a novel filterable alphaproteobacterium isolated from soil using a gel-filled microwell array device
Source: PLoS One. 2024 Jun 10;19(6):e0304366. doi: 10.1371/journal.pone.0304366 (PMC11164329; doi:10.1371/journal.pone.0304366)
Supplement: S1 Appendix — (PDF) [file pone.0304366.s001.pdf]

Roseiterribacter gracilis gen. nov., sp. nov., a novel filterable alphaproteobacterium isolated from soil using a gel-filled microwell array device

- Supporting Figures -

Ryosuke Nakai[a*], Hiroyuki Kusada[b], Fumihiro Sassa[c], Ayaka Makino[a†], Susumu Morigasaki[d], Hisayoshi Hayashi[d], Naoki Takaya[d,e], Hideyuki Tamaki[b,d,e*]

Affiliation:

[a] Bioproduction Research Institute, National Institute of Advanced Industrial Science and Technology (AIST), 2-17-2-1, Tsukisamu-higashi, Toyohira-ku, Sapporo, Hokkaido 062-8517, Japan
[b] Bioproduction Research Institute, National Institute of Advanced Industrial Science and Technology (AIST), 1-1-1 Higashi, Tsukuba, Ibaraki 305-8566, Japan
[c] Department of Electronics, Graduate School of Information Science and Electrical Engineering, Kyushu University, 744, Motooka, Nishi-ku, Fukuoka 819-0395, Japan
[d] Institute of Life and Environmental Sciences, University of Tsukuba, 1-1-1, Tennodai, Tsukuba, Ibaraki 305-8572, Japan
[e] Microbiology Research Center for Sustainability, University of Tsukuba, 1-1-1, Tennodai, Tsukuba, Ibaraki 305-8572, Japan

[*]Address correspondence to Ryosuke Nakai, nakai-ryosuke@aist.go.jp and Hideyuki Tamaki, tamaki-hideyuki@aist.go.jp

[†]Present address: Research Institute of Energy, Environment and Geology, Hokkaido Research Organization (HRO), Kita 19 Nishi 12, Sapporo 060-0819, Japan

Table of Contents

Supporting Figures 2

S1 Figure 2

S2 Figure 3

S3 Figure 4

S4 Figure 5

S5 Figure 6

S6 Figure 7

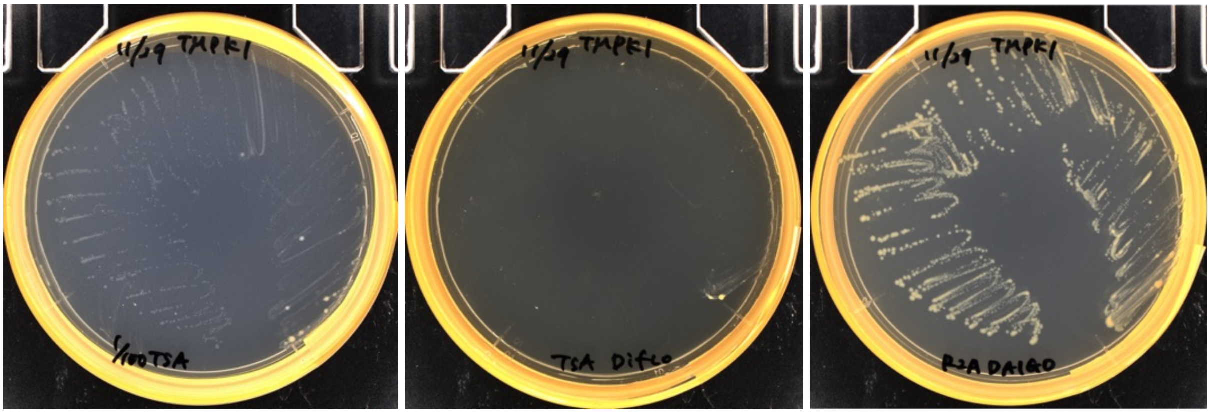

1/100 TSA TSA R2A

S1 Fig. Images of TMPK1^T colonies on 1/100 TSA medium (*left*), undiluted standard TSA medium (*central*), and R2A medium (*right*).

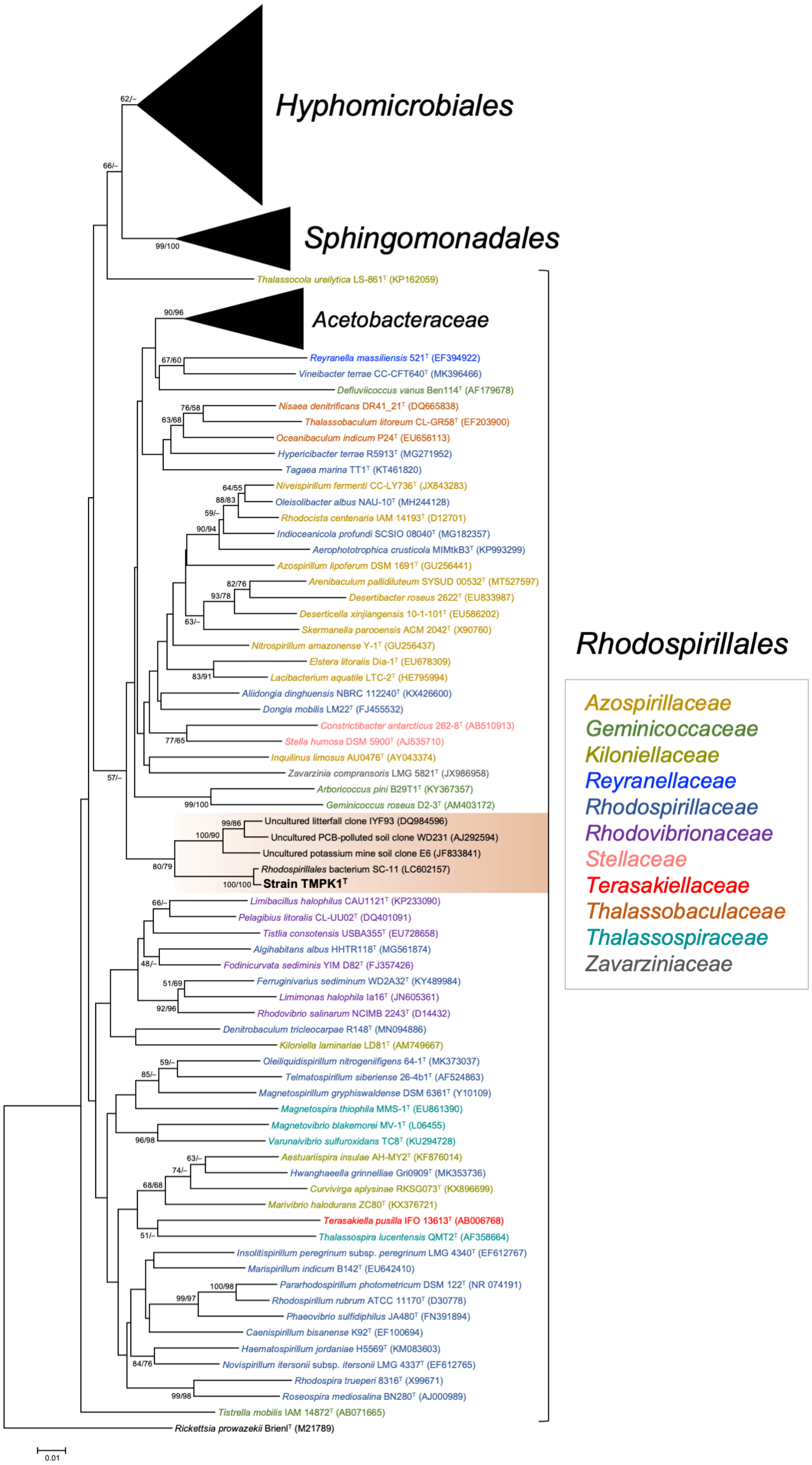

S2 Fig. Phylogenetic tree of strain TMPK1^T and members of the order *Rhodospirillales* and other orders based on the 16S rRNA gene sequences. The evolutionary history was inferred using the Neighbor-Joining (NJ) method. There were a total of 962 positions in the final dataset. Bootstrap values of >50% are shown from left to right for NJ and maximum-likelihood (ML) trees with the same sequence data set (1,000 replications for NJ and 100 replications for ML). Evolutionary analyses were conducted in MEGA7.

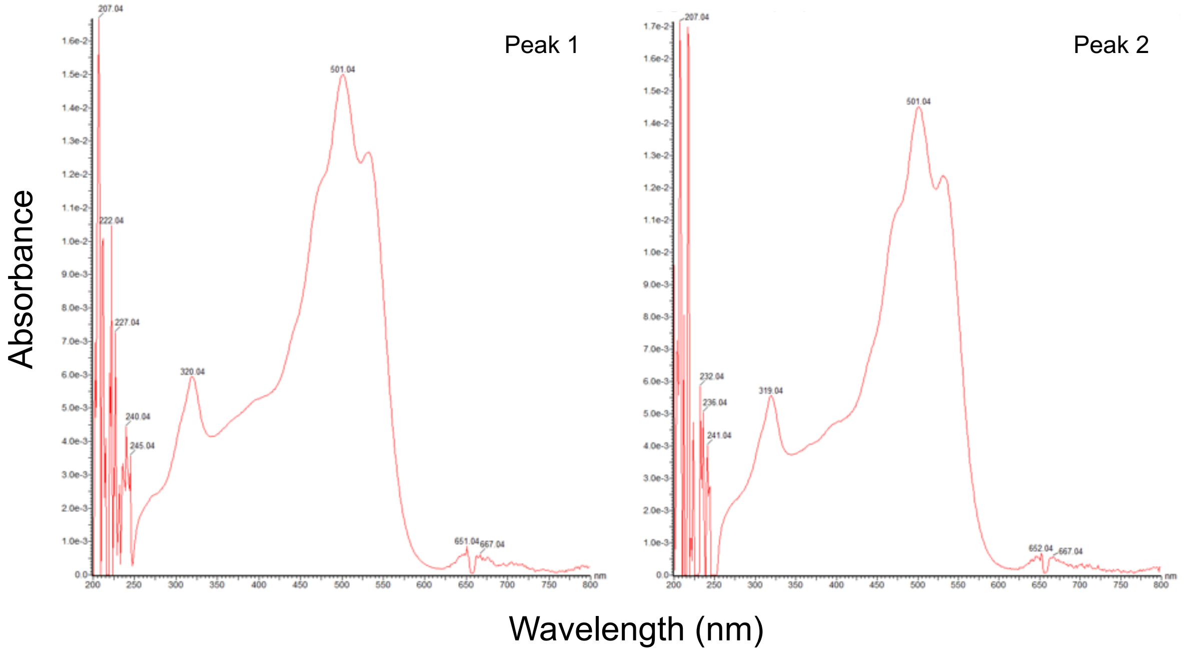

S3 Fig. Two absorbance peaks of extracted pigments of strain TMPK1^{T}. Cells destined for pigment analysis were cultivated under light condition.

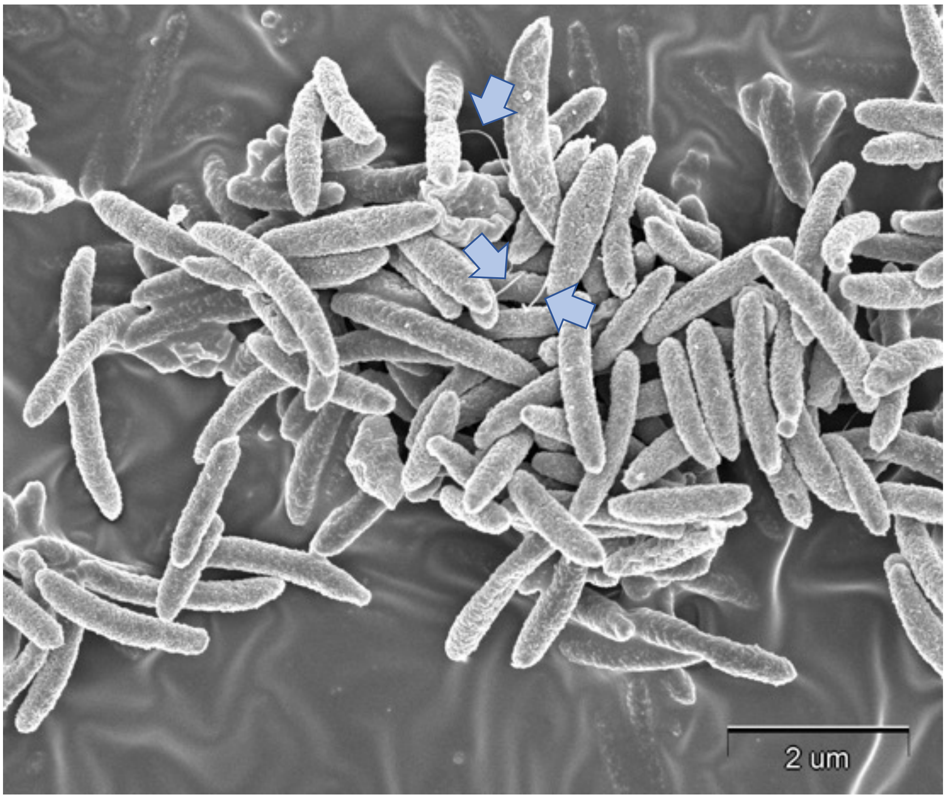

S4 Fig. Scanning electron micrograph of cells of strain TMPK1^{T}. Some cells had flagellum-like structures (light blue arrows). Scale bar, 2 μm.

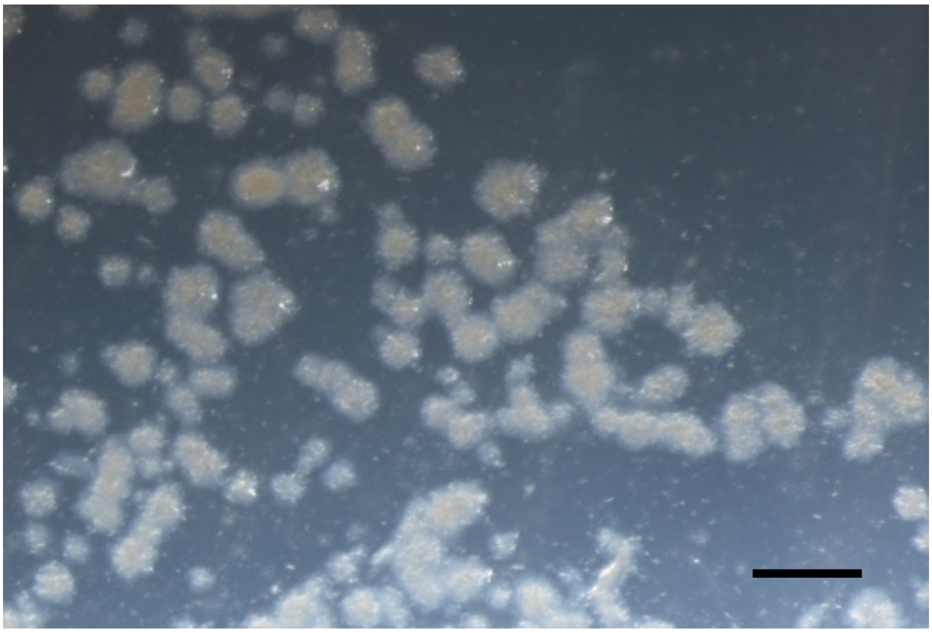

S5 Fig. Images of colonies of strain TMPK1^T grown on R2A medium supplemented with Tween 40. Scale bar, 1 mm.

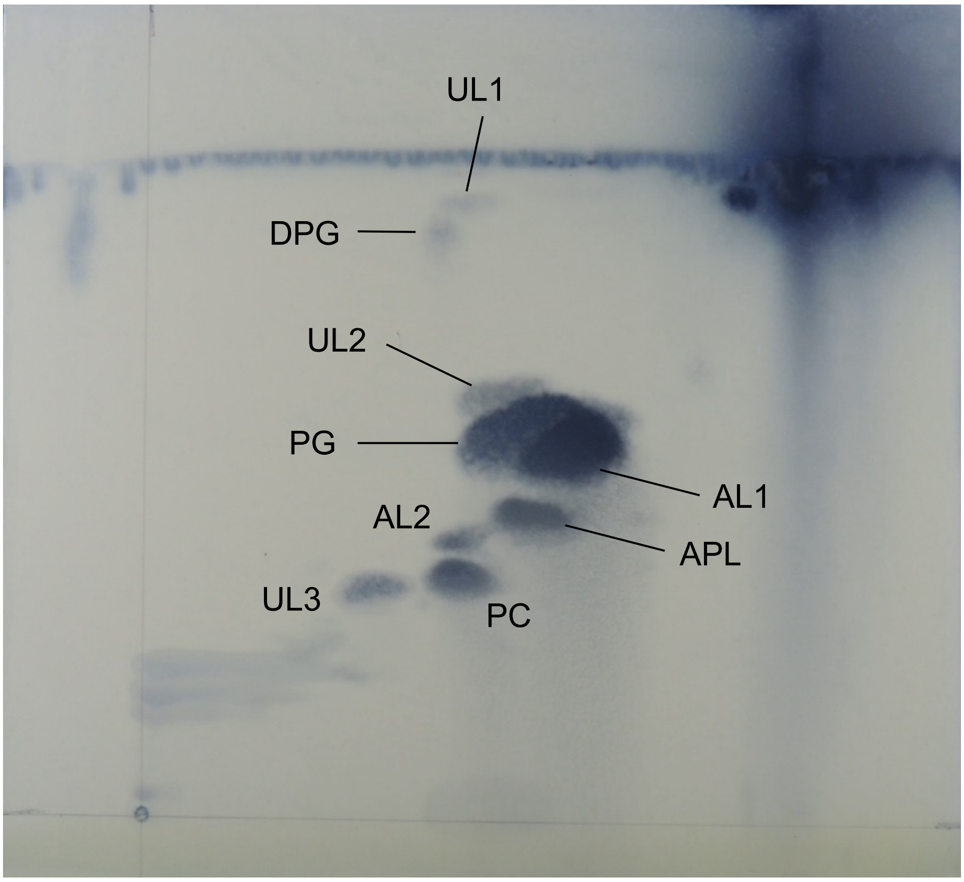

S6 Fig. Polar lipids of strain TMPK1^T after separation by two-dimensional HPTLC. PG, phosphatidylglycerol; AL1, unidentified amino lipid 1; DPG, diphosphatidylglycerol; PC, phosphatidylcholine; UL1–3, unidentified polar lipids; AL2, unidentified amino lipid 2; APL, unidentified amino phospholipid.
